# Hexvix blue light fluorescence cystoscopy – a promising approach in superficial bladder tumors diagnosis

**Published:** 2008-08-15

**Authors:** Geavlete B., Mulţescu R., Georgescu D., Geavlete P.

**Affiliations:** * Department of Urology, „Saint John” Emergency Clinical Hospital, Bucharest, Romania

## Abstract

**Introduction:** Nowadays, Hexvix blue light cystoscopy (BLC) represents an increasingly acknowledged diagnostic method for patients with bladder cancer. The aim of our study was to establish the place of this procedure in superficial bladder tumors diagnosis and to compare it with standard white light cystoscopy (WLC).

**Material and methods:** Between December 2007 and January 2008, WLC and BLC were performed in 20 cases. Transurethral bladder resection (TURB) was performed for all apparent detected lesions. The patients diagnosed with superficial bladder tumors have been followed-up after 3 months by WLC and BLC. The control group included the same number of consecutive patients with superficial bladder tumors, diagnosed only by WLC, which underwent the same treatment and follow-up protocol as the study group.

**Results:** WLC identified 30 suspicious lesions (28 pathologically confirmed), while BLC identified 41 apparent tumors (39 pathologically confirmed). So, from the total number of 40 tumors with positive histology, WLC correctly diagnosed 70% of them, with a rate of 6.7% false-positive results, while BLC diagnosed 97.5%, however presenting a 4.9% rate of false-positive results. 17 cases of the study group diagnosed with superficial bladder tumors were followed. The tumor recurrence rate after 3 months was 5.9% for the study group and 23.5% for the control group.

**Conclusions:** Hexvix fluorescence cystoscopy is a valuable diagnostic method, with considerably better results by comparison to WLC. The improved diagnostic may have a significant impact upon the recurrence rate.

## Introduction

Bladder cancer represents a common malignancy, with a severely high recurrence rate. Despite the fact that WLC is still regarded as the gold-standard diagnostic method for superficial bladder tumors [**1**], small papillary (Ta, T1) or flat (CIS) urothelial lesions can be easily overlooked, thus leading to a significant increase of the recurrence rate [**2**].

So, while searching for a more sensitive diagnostic tool, photodynamic diagnosis (PDD) emerged as a promising solution.

The aminolevulinic acid (ALA) was among the first products used for PDD, but only after the introduction of its improved ester, hexyl aminolevulinate (HAL - Hexvix®) did the method acquire a substantial acknowledgement in practical use.

The EAU Guidelines state that fluorescence cystoscopy, performed in blue-light and using a porphyrin-based photo sensitizer, may help discovering suspicious areas, which can hardly be detected in white-light [**1**]. Consequently, TURB under fluorescence guidance seems to reduce the risk of tumor recurrence [**3**]. 

In our country, Hexvix fluorescence cystoscopy was performed as a national premiere in the Department of Urology of “Saint John” Emergency Clinical Hospital in December 2007.

## Materials and Methods:

Between December 2007 and January 2008, 20 consecutive cases, 14 men and 6 women, with a mean age of 66 years (range 36 to 78) with hematuria and positive urinary cytology have been investigated in our Clinical Department.

A standard investigative protocol was applied in all cases and included: general clinical examination, blood tests, urine culture, abdominal ultrasonography and IVP. No imagistic evidence of upper urinary tract disease has been found. 

Hexvix bladder instillation (100mg dissolved in 50mL phosphate buffer solution, 8mmol) was performed using a 14 Ch bladder catheter, after complete voiding, at least one hour prior to cystoscopy. The catheter was removed after instillation (except in 1 case with urinary incontinence, when it was simply clamped). Patients were instructed not to void and to repeatedly change position in order to insure the contact of the entire bladder urothelium with hexaminolevulinate. 

The necessary equipment for BLC consisted of:

• a high-power endoscopic light source with integrated excitation filter (wavelength 380-450 nm), which passes primarily blue light (D-light-C Storz system);

• a special light cord;

• a high sensitivity version of the endoscopic camera, displaying a special “fluorescence mode”;

• a foot pedal which allows convenient switching between white and blue light;

• a standard color monitor;

• a Storz rigid cystoscope with an integrated filter in the eyepiece.

All procedures have been performed under spinal anesthesia. 

**Figure 1:**
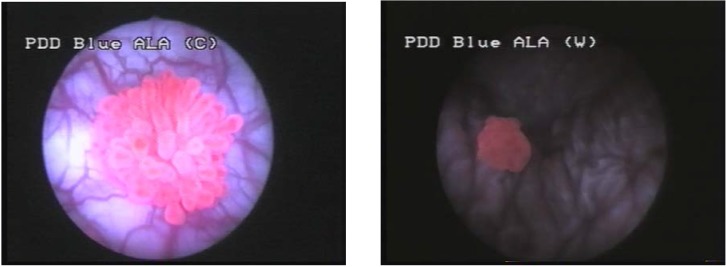
Hexvix induced fluorescence of bladder urothelial tumors in blue light

The first step of the endoscopic procedure was represented by repeated washing of the bladder, aiming to evacuate the Hexvix solution, consequently avoiding the excessive fluorescence of the bladder content. Thus, the contrast required for small lesions’ detection was significantly improved. The second step included careful WLC, resulting in a bladder map of the suspicious lesions. Standard cystoscopy was followed by BLC, and the distinctively fluorescent lesions were also mapped (**Fig. 1**).

A comparison of some uncertain lesions on the two bladder charts was obtained by repeated switching from white to blue light, and backwards. 

TURB was performed for all the suspected lesions established by the two types of cystoscopy. The histological analysis emphasized a “pathological” bladder chart for each patient. A comparison between the three bladder maps was performed, in order to establish the accuracy of each diagnostic method. 

The patients diagnosed with superficial bladder tumors have been followed-up after 3 months by WLC and BLC. The control group included the same number of consecutive patients with superficial bladder tumors, diagnosed only by WLC, which underwent the same treatment and follow-up protocol as the study group. 

## Results

The cystoscopy and pathological results described 4 different groups of patients.

Group I included cases with identical bladder maps according to WLC and BLC, and entirely confirmed by the pathological examination. 

This group included 10 cases (50%) in which 18 tumoral lesions were identified by both diagnostic tools (1 CIS, 9 pTa, 7 pT1 and 1 pT2). There were no false-positive lesions among these cases, according to both methods. 

Group II included the cases diagnosed with bladder cancer by WLC and in which BLC showed at least one supplementary bladder tumor.

**Figure 2:**
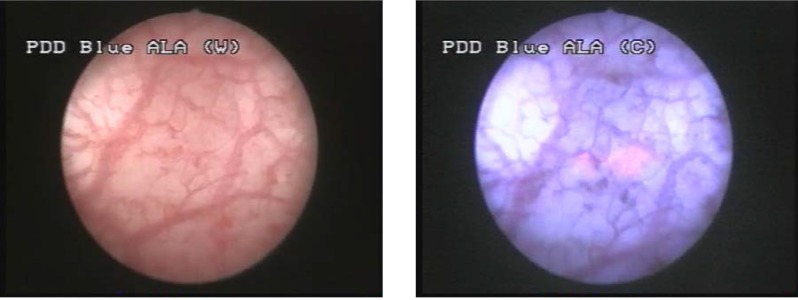
Comparative aspect in WLC (left) and BLC (right) of two pTa urothelial bladder tumors

**Figure 3:**
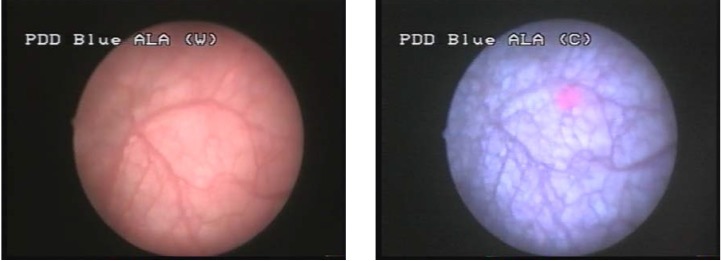
pTa urothelial tumor not visible in white light but with specific fluorescence in blue light

This group consisted of 7 patients (35%) in which WLC described 10 pathologically confirmed tumors (4 pTa, 5 pT1 and 1 pT2). BLC identified 9 additional lesions, 8 of which pathologically confirmed (2 CIS and 6 pTa) (**Fig. 2**, **3**). In one case, a pTa tumor described by WLC, presented no fluorescence in BLC.

**Figure 4:**
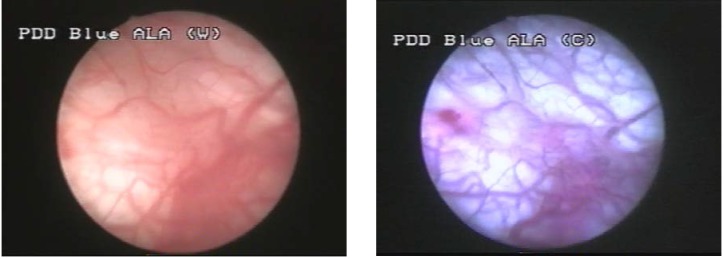
CIS not visible in white light but with specific fluorescence in blue light

**Figure 5:**
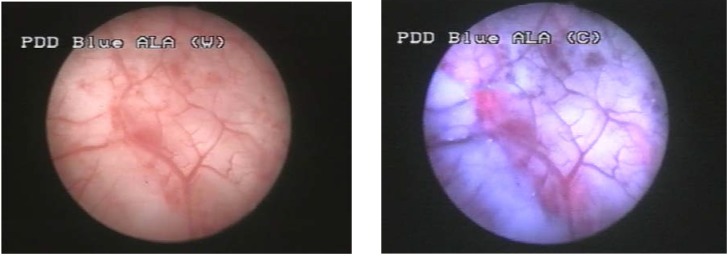
Another case with CIS with not specific aspect in WLC but fluorescent in BLC

Group III consisted of 2 cases (10%), in which WLC found no tumoral lesion and BLC described 5 CIS lesions, 4 of which being confirmed by pathology (**Fig. 4**, **5**).

**Figure 6:**
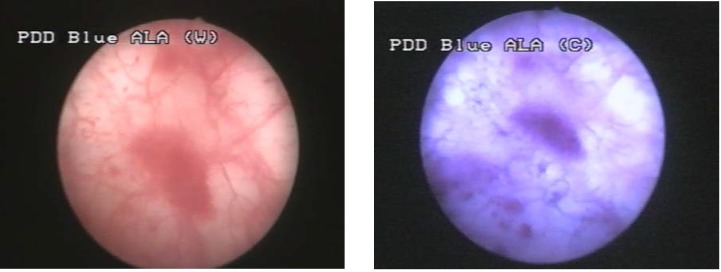
Suspicious flat lesion in WLC, with no fluorescence in BLC and no pathological confirmation

Group IV included one case (5%), in which WLC showed two apparently flat lesions, with no fluorescence in BLC and no pathological confirmation (**Fig. 6**). 

Summarizing these data, a total number of 40 tumors (7 CIS, 19 pTa, 12 pT1, 2 pT2) have been confirmed as malignant by pathology in 19 cases (95%).

The sensitivity of WLC was 70%, 28 of the actual 40 tumors being correctly diagnosed (1 CIS, 13 pTa, 12 pT1 and 2 pT2). The false positive rate of this method was 6.7% (2 out of 30 resected suspicious lesions turned out to be benign).

Forty-one suspicious tumoral lesions were identified during BLC, of which 39 were confirmed by the pathology exam (7 CIS, 18 pTa, 12 pT1, 2 pT2). The false positive results were represented by 2 CIS suspicious lesions (4.9%). One pTa tumor described by WLC was not diagnosed by BLC. Therefore, this diagnostic method described a sensitivity of 97.5% (39 out of the 40 pathologically confirmed tumors have also been emphasized in blue light). 

There were no complications related to Hexvix-BLC. 

Regarding the follow-up, both patients with pT2 tumors (which underwent radical cystectomy) and the one with no pathologically confirmed malignancy were excluded from the study group. Among the remaining 17 patients, after re-TURB, 1 case of recurrence was identified, with a pathologically confirmed CIS lesion. In the control group were diagnosed 4 cases of recurrence (5 tumors, of which 2 pTa and 3 CIS). So, the tumor recurrence rate was 5.9% for the study group, and 23.5% for the control group.

## Commentaries

BLC is a diagnostic method which emerged from the constant need to improve the efficacy of standard WLC.

The first attempts in PDD involved tetracycline fluorescence (1957), HpD, a haematoporphyrin derivate (1975), Photofrin (1987) and 5-ALA, a precursor of photoactive porphyrin (1992) [**4**]. 

ALA was the first topical agent used for PDD. Nowadays, it has been replaced by a more potent lipophilic derivate, HAL–Hexvix, an improved ester of the aminolevulinic acid, which provides the benefits of increased selectivity, brighter fluorescence and shorter instillation time [**5**, **6**].

The basis of Hexvix cystoscopy is represented by the increased preferential accumulation of the photoactive porphyrin in the neoplastic tissue, resulting in red fluorescence emitting tumors (including some of the previously undetected ones) [**7**].

The accuracy of BLC depends on a number of important practical aspects concerning the cystoscopy technique and the specific issues related to it. One of the most important goals is to improve the specificity of the method by reducing the number of unnecessary biopsies. There are some distinctive causes related to false-positive fluorescence

**Figure 7:**
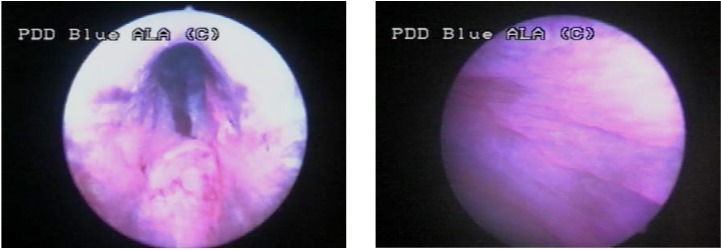
Normal fluorescence of the prostatic urethra (left) and bladder neck (right)

One of them is represented by the usually fluorescent appearance of the prostatic urethra, bladder neck and the ureteral orifices (**Fig. 7**), unrelated to malignancy.

**Figure 8:**
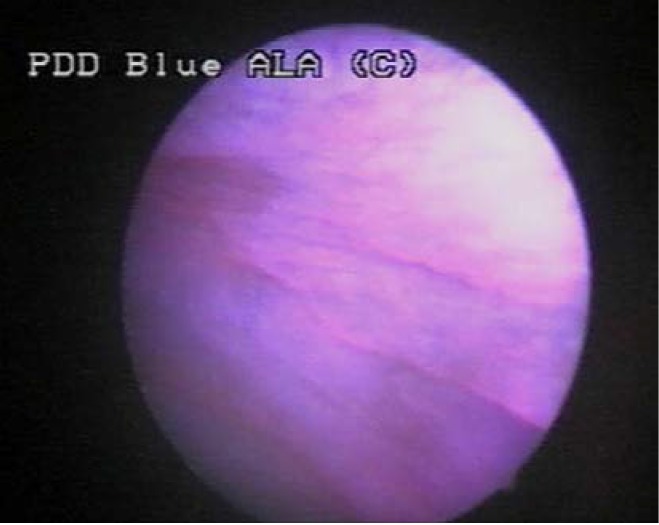
False positive fluorescence of the normal urothelium in tangential view

Also, tangential view in blue light may create a false fluorescence of the normal urothelium (**Fig. 8**). In order to avoid this problem the bladder must be fully distended (thus eliminating the presence of the mucosal folds) and the lesions must be directly illuminated.

The false fluorescence may be emphasized by pressing the concerned area with the resection loop. Such lesions in which fluorescence fades in this manner are usually benign and therefore resection is unnecessary.

Bladder inflammation may determine increased fluorescence of the mucosa and consequently, false-positive results [**8**]. That is why BLC should not be performed any sooner than 3 months after BCG (Bacillus Calmette-Guerin) or chemotherapy intravesical instillations. 

The false positive results are much related to the aggressiveness of certain urologists while performing bladder biopsies, due to their tendency to resect any remotely suspicious areas. A review of the literature emphasized a rate of unnecessary biopsies of 13 to 39% [**5**, **8**].

**Figure 9:**
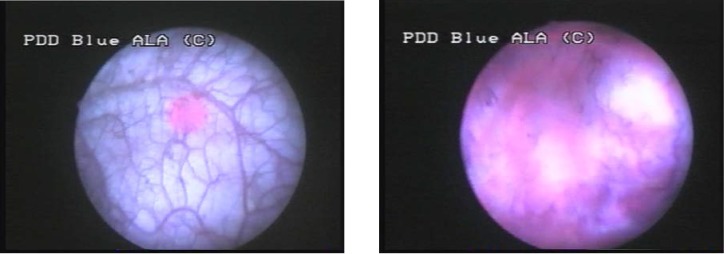
Sharp margins of a malignant lesion (left) by comparison to diffuse aspect of a false-positive fluorescence secondary to inflammation (right)

The margins of a fluorescent malignant lesion have to appear quite sharp and well separated from the surrounding regions (**Fig. 9**).

Blue light is highly absorbed by blood and clots; therefore, resection related bleeding may result in poor visualization and diagnostic accuracy. TURB is bound to take place only after completing the 2 types of cystoscopy [**8**].

The exclusion criteria for patients taken into consideration for Hexvix BLC are represented by allergy to HAL or related compounds, pregnancy, lactation, intravesical instillations.

In a multicentric study which included 298 patients, Fradet and Grossman compared Hexvix - BLC and WLC regarding the detection of CIS. In 58 cases with 113 CIS lesions, BLC detected 104 (92%), while WLC only established the presence of 77 (68%). Therefore, the authors concluded that Hexvix - BLC is able to diagnose CIS lesions that may be missed by WLC [**7**].

Frampton and Plosker mention two European, multicentric, phase III trials, which analyzed HAL cystoscopy as an adjunct procedure to standard cystoscopy in patients with known or suspected bladder cancer. According to one trial, HAL cystoscopy detected 96% of the patients with CIS (28% more patients with CIS than standard cystoscopy). In the second trial, 17% of patients were selected to receive a more complete treatment, due to the improved tumor detection rate following HAL cystoscopy [**9**].

Grossman evaluated 311 patients in another multicenter study, HAL fluorescence cystoscopy being compared with WLC concerning the detection of Ta and T1 papillary lesions. In 29% of the cases, Hexvix - cystoscopy detected at least 1 more Ta and T1 papillary tumor than WLC. Detection rates for HAL and standard cystoscopy were 95% vs. 83% for Ta tumors, respectively 95% vs. 86% for T1 tumors [**8**].

Loidl and Schmidbauer accomplished a prospective controlled study, in which they analyzed a within-patient parallel between flexible HAL cystoscopy and standard flexible cystoscopy, HAL rigid and standard white light rigid cystoscopy. HAL fluorescence flexible cystoscopy compared to HAL rigid cystoscopy showed almost equivalent results in detecting papillary and flat lesions in bladder cancer patients, both procedures being superior to standard white light flexible cystoscopy [**2**].

Most of the BLC side-effects are related to the endoscopic procedure rather than the HAL instillation, with no significant differences from WLC (dysuria, hematuria, bladder spasm, and bladder pain) [**4**].

Due to the fact that 5-ALA and HAL do not penetrate much deeper than 1mm, fluorescence cannot indicate the invasion depth. So, a second TURB should be performed in T1 tumors, in order to rule out muscle invasion [**4**].

It has been shown that recurrence at any site in the bladder at the first follow-up cystoscopy after TUR is one of the most important prognostic factors for time to progression. Therefore, PDD might be most useful for patients with primary tumors [**4**].

It is quite obvious that tumors shortly diagnosed after TURB are mostly lesions overlooked during the primary procedure rather then newly developed malignancies. This is the rationale for trying to improve the short term recurrence rate and the prognosis by superior diagnostic accuracy.

Denziger and Burger performed a comparative analysis on 191 cases with superficial bladder tumors diagnosed either by WLC or BLC. The recurrence rates among these patients were 44% in the WLC arm versus 16% in the BLC arm. Moreover, the residual tumors rate at 6 weeks after the primary TURB was 25.2% and respectively 4.5% [**10**].

Similar results have been established by Daniltcenko and Riedl, describing recurrence rates of 16% in cases investigated by BLC and 41% in patients diagnosed by standard protocol. The differences between the recurrence rates tend to decrease in time. At 2, 12, 36 and 60 months from the initial TURB the rates were 41%, 61%, 73% and 75% in the WLC arm versus 16%, 43%, 59% and 59% in the BLC arm [**11**].

In the literature data, results concerning secondary diagnosed lesions are rather contradictory. Filbek and Pichlmeir discovered no heterotopic residual tumors during the second look TURB. On the other hand, Riedl and Daniltcenko emphasized an almost similar proportion of orthotopic and heterotopic residual malignancies [**12**, **13**].

## Conclusions

Hexaminolevulinate (HAL–Hexvix) fluorescence cystoscopy proves to be a powerful diagnostic method in superficial bladder cancer, with more effective imaging, higher detection rates and improved sensitivity by comparison to WLC.

Patients with Ta, T1 and especially CIS are the main beneficiaries of this technique, as it provides them with better chances for a complete TURB.

The improved accuracy of BLC leads to a significantly reduced recurrence rate, especially during the short and medium-term follow-up. 

The impact of Hexvix-BLC upon the prognostic and the survival rates in patients with superficial bladder tumors should represent the main objective for future large and multicenter studies. 
